# Tantalum Surgical Clip Presenting As an Intraorbital Foreign Body

**DOI:** 10.18502/jovr.v18i1.12734

**Published:** 2023-02-21

**Authors:** George P. Kung, Jeremy D. Clark, Austin Gerber, Niloofar Piri

**Affiliations:** ^1^Department of Ophthalmology, Saint Louis University School of Medicine, St. Louis, Missouri; ^2^Department of Ophthalmology and Visual Sciences, Kentucky Lions Eye Center, University of Louisville, Louisville, Kentucky

##  PRESENTATION

**Figure 1:**
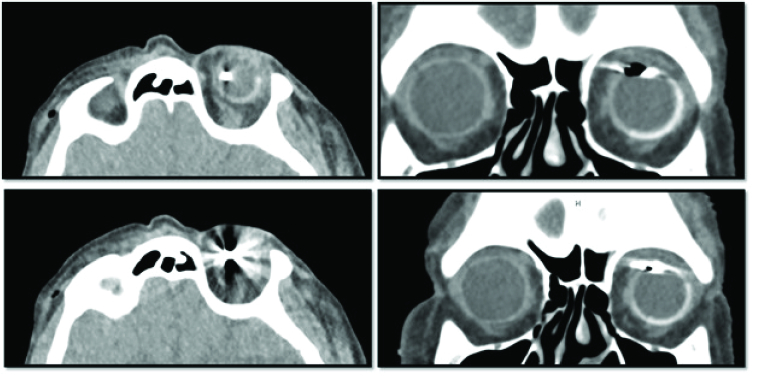
Orbital CT scan. Axial sections with suspected intraocular hyperdense metallic foreign body (Left panel). Large metallic foreign body with irregular borders complicated by streak artifact and air track superonasally embedding into the sclera (Right panel). CT, computed tomography.

An 87-year-old female presented to the emergency room after she was partially run over by a truck and sustained multiple injuries including skull and facial trauma. Facial bones CT scan was significant for a large, metallic intraorbital foreign body on the left side [Figure 1]. Per radiology, an intraocular foreign body (IOFB) could not be ruled out. Ophthalmology department was consulted to evaluate. She had a history of scleral buckling in the left eye for rhegmatogenous retinal detachment in the 1990s, with chronic mild low vision at baseline.

Her near corrected visual acuity was 20/20 OD and 20/60 OS; intraocular pressures were 14 OD, 11 mmHg OS. A relative afferent pupillary defect was present on the left. Examination revealed left upper lid hematoma, lower lid ecchymosis, deep laceration above eyebrow, superior subconjunctival hemorrhage, and pseudophakia. Fundus exam on the left revealed no vitreous hemorrhage, 360 high buckle effect, temporal cryopexy scars, and small hemorrhage on the buckle. No intraocular penetration site was seen, and foreign body appeared to be intraorbital only.

**Figure 2:**
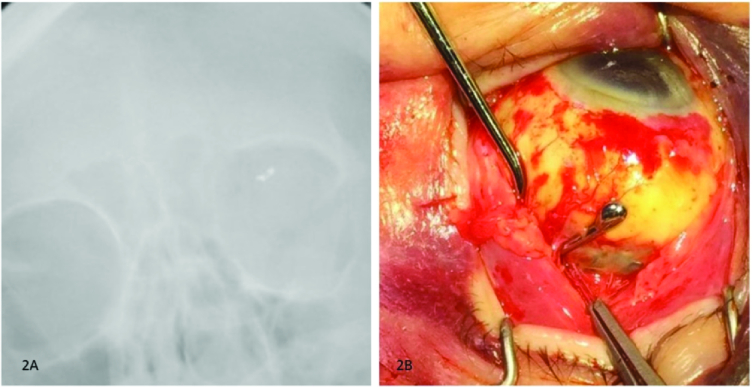
(A) Intraoperative X-ray demonstrating metallic foreign body in left orbit to be symmetric surgical clip. (B) Metallic surgical clip used to secure buckle located superonasally.

Laceration was the suspected entry site. Clinical exam was not concerning for IOFB, but given the large, and irregular shape of the foreign body in addition to embedding on the globe with air track, the decision was made to proceed with exploratory orbitotomy and foreign body removal.

Superior orbitotomy through upper lid crease was performed with opening of the septum. Exploration and irrigation failed to retrieve any foreign body. Intraoperative skull X-ray was performed, which revealed the presence of a small metallic foreign body in the superonasal orbit in a regular shape similar to a surgical clip [Figures 2A & 2B].

Surgical plan was changed, and superior 120º peritomy was performed with isolation of the superior rectus muscle. Metallic foreign body was revealed to be a tantalum surgical clip. Upon contact with the retina surgeon's office, it was confirmed to be a non-magnetic tantalum clip used to secure the scleral buckle.

##  DISCUSSION

While ophthalmologists have moved onto the usage of sutures or silicone sleeves to secure scleral buckles in the past two decades, tantalum clips were a common option in historical scleral buckle surgery.^[1]^

The properties of tantalum posed a unique diagnostic challenge in this case. While radiopaque on X-rays, tantalum has the disadvantage of producing streak artifacts on CT.^[1,2,3]^ The irregular margins of the foreign body produced by the artifact on CT made it difficult to not only see the shape of the clip but also localize its location. Plain radiographs are not a typical part of foreign body workup due to their underestimation of common radiolucent foreign bodies such as wood or plastic.^[4,5]^ Had an X-ray been considered in the context of the patient's surgical history and artifact on CT, the surgical clip likely would have been identified earlier and surgery been avoided.

In summary, foreign bodies can present a complex problem when initial diagnostic imaging is uncertain. In patients with a historical scleral buckle procedure, consider the presence of tantalum clips as a possibility.

##  Financial Support and Sponsorship

None.

##  Conflicts of Interest

None.
